# The impact of digital media on children’s intelligence while controlling for genetic differences in cognition and socioeconomic background

**DOI:** 10.1038/s41598-022-11341-2

**Published:** 2022-05-11

**Authors:** Bruno Sauce, Magnus Liebherr, Nicholas Judd, Torkel Klingberg

**Affiliations:** 1 Department of Biological Psychology, Vrije Universiteit Amsterdam, Amsterdam, The Netherlands; 2 Department of General Psychology: Cognition, University Duisburg-Essen, Duisburg, Germany; 3 Department of Neuroscience, Karolinska Institutet, Solna, Sweden

**Keywords:** Behavioural genetics, Human behaviour

## Abstract

Digital media defines modern childhood, but its cognitive effects are unclear and hotly debated. We believe that studies with genetic data could clarify causal claims and correct for the typically unaccounted role of genetic predispositions. Here, we estimated the impact of different types of screen time (watching, socializing, or gaming) on children’s intelligence while controlling for the confounding effects of genetic differences in cognition and socioeconomic status. We analyzed 9855 children from the USA who were part of the ABCD dataset with measures of intelligence at baseline (ages 9–10) and after two years. At baseline, time watching (r = − 0.12) and socializing (r = − 0.10) were negatively correlated with intelligence, while gaming did not correlate. After two years, gaming positively impacted intelligence (standardized β =  + 0.17), but socializing had no effect. This is consistent with cognitive benefits documented in experimental studies on video gaming. Unexpectedly, watching videos also benefited intelligence (standardized β =  + 0.12), contrary to prior research on the effect of watching TV. Although, in a posthoc analysis, this was not significant if parental education (instead of SES) was controlled for. Broadly, our results are in line with research on the malleability of cognitive abilities from environmental factors, such as cognitive training and the Flynn effect.

## Introduction

Rapid technological progress has shaped modern childhood. Time spent with digital devices has increased dramatically since the start of our twenty-first century^1,2^ and is now a large portion of a child’s free time^3^. Children aged 8–12, for example, spend an average of 4–6 h with a screen each day watching videos, gaming, and socializing^1,4^. Digital media bring a variety of new experiences that could have both benefits and drawbacks to the developing minds of children^5^. This topic is a source of heated public debates^6^ and deserves careful attention from policymakers and researchers.

Our focus here is on the impact of screen time on intelligence: the ability to learn effectively, think rationally, understand complex ideas, and adapt to new situations. Intelligence is one of the most important and well-studied psychological traits and encompasses multiple cognitive processes: attention, working memory, spatial manipulation, processing speed, reasoning, reading comprehension, long-term memory, and others^7,8^. Scores in measures of intelligence are highly predictive of a person’s future, including happiness, longevity, income, and educational achievement^9^.

Research so far shows mixed consequences of screen time on cognitive abilities related to intelligence^10–13^. Effect sizes typically range from − 0.20 to + 0.20 depending on the study, though there are cases with more extreme values. This picture is further complicated because different types of screen activities might have distinct effects on intelligence^3,13^. For example, a cross-sectional study^13^ found that children who spent more time playing video games (in the high tertile) had a positive association with intelligence compared to low tertile (β =  + 0.12). However, children in the high tertile for TV watching (β = − 0.99), video watching (β = − 1.05), and social media (β = − 0.79) had lower measures of cognition compared to children in the low tertile for each variable.

Other studies have focused on the impact of screen time on school performance—a trait that is not equivalent to intelligence but that shares a strong causal relationship in both directions^14^. A large longitudinal study found that watching TV and using computers both led to worse school performance, but there were no short- or long-term effects from playing video games^15^. Cross-sectional studies also report negative effects of television viewing on attention and learning^16,17^, while a large meta-analysis found that video games do not negatively impact school performance^18^.

Adding to the complexity of the screen time question: lack of perseverance is a frequently described mediator/confounder, especially in terms of problematic usage behavior^19^. Lack of Perseverance is defined as a failure in maintaining focus on a boring or difficult task^20^ and is associated with cognitive functions^21^ and school achievement^22^.

Video games seem to be a unique type of digital activity. Empirically, the cognitive benefits of video games have support from multiple observational and experimental studies^23–25^. Their benefits to intelligence and school performance make intuitive sense and are aligned with theories of active learning and the power of deliberate practice^26,27^. There is also a parallel line of evidence from the literature on cognitive training intervention apps^28,29^, which can be considered a special (lab developed) category of video games and seem to challenge some of the same cognitive processes. Though, like for other digital activities, there are contradictory findings for video games, some with no effects^30,31^ and negative effects^32,33^.

The contradictions among studies on screen time and cognition are likely due to limitations of cross-sectional designs, relatively small sample sizes, and, most critically, failures to control for genetic predispositions and socio-economic context^10^. Although studies account for some confounding effects, very few have accounted for socioeconomic status and none have accounted for genetic effects. This matters because intelligence, educational attainment, and other cognitive abilities are all highly heritable^9,34^. If these genetic predispositions are not accounted for, they will confound the potential impact of screen time on the intelligence of children. For example, children with a certain genetic background might be more prone to watch TV and, independently, have learning issues. Their genetic background might also modify the impact over time of watching TV. Genetic differences are a major confounder in many psychological and social phenomena^35,36^, but until recently this has been hard to account for because single genetic variants have very small effects. Socioeconomic status (SES) could also be a strong moderator of screen time in children^37^. For example, children in lower SES might be in a less functional home environment that makes them more prone to watch TV as an escape strategy, and, independently, the less functional home environment creates learning issues. Although SES is commonly assumed to represent a purely environmental factor, half of the effect of SES on educational achievement is probably genetically mediated^38,39^—which emphasizes the need for genetically informed studies on screen time.

Here, we estimated the impact of different types of screen time on the change in the intelligence of children in a large, longitudinal sample, while accounting for the critical confounding influences of genetic and socioeconomic backgrounds. In specific, we had a strong expectation that time spent playing video games would have a positive effect on intelligence, and were interested in contrasting it against other screen time types. Our sample came from the ABCD study (http://abcdstudy.org) and consisted of 9855 participants aged 9–10 years old at baseline and 5169 of these followed up two years later.

For our genetic measures, we used polygenic scores: an index that summarizes the best current estimates of additive genetic influences towards a particular trait^40^. Polygenic scores have a variety of useful applications, opening up the possibility of controlling for and investigating relationships that would have been practically impossible otherwise^35^. Recently, a large, genome-wide association study with 1.1 million people was able to estimate the effect of thousands of genetic regions on cognitive performance, educational attainment, and mathematical ability^34^. The combination of these regions was able to explain 11–13% of the variance in educational attainment and 7–10% of the variance in cognitive performance. With that tool available, here we were able to sum the reported effect sizes of all available genetic markers to create polygenic scores for cognitive performance (cogPGS) for each child in our sample—providing our study with a much greater statistical power than typical in the past.

We estimated the effect of screen time when already accounting for the potential confounders of cogPGS and SES on intelligence at age 9–10 and the change in the same children two years later (while also controlling for age at each time point, site of data collection, and genetic population stratification). In addition, we performed gender analyses to verify previously reported differences in screen time between boys and girls. Finally, we performed a mediation analysis with lack of perseverance on the role of screen time in intelligence.

## Results

### Measuring intelligence and testing its equivalence at both time points

Data from 9855 children recruited by the ABCD study (https://abcdstudy.org) were included. Table 1 shows the Pearson correlations between all five cognitive tasks used here to represent intelligence: Picture Vocabulary Task, Flanker Task, Oral Reading Recognition Task, Rey Auditory Verbal Learning Task, and Little Man Task.

**Table 1:**
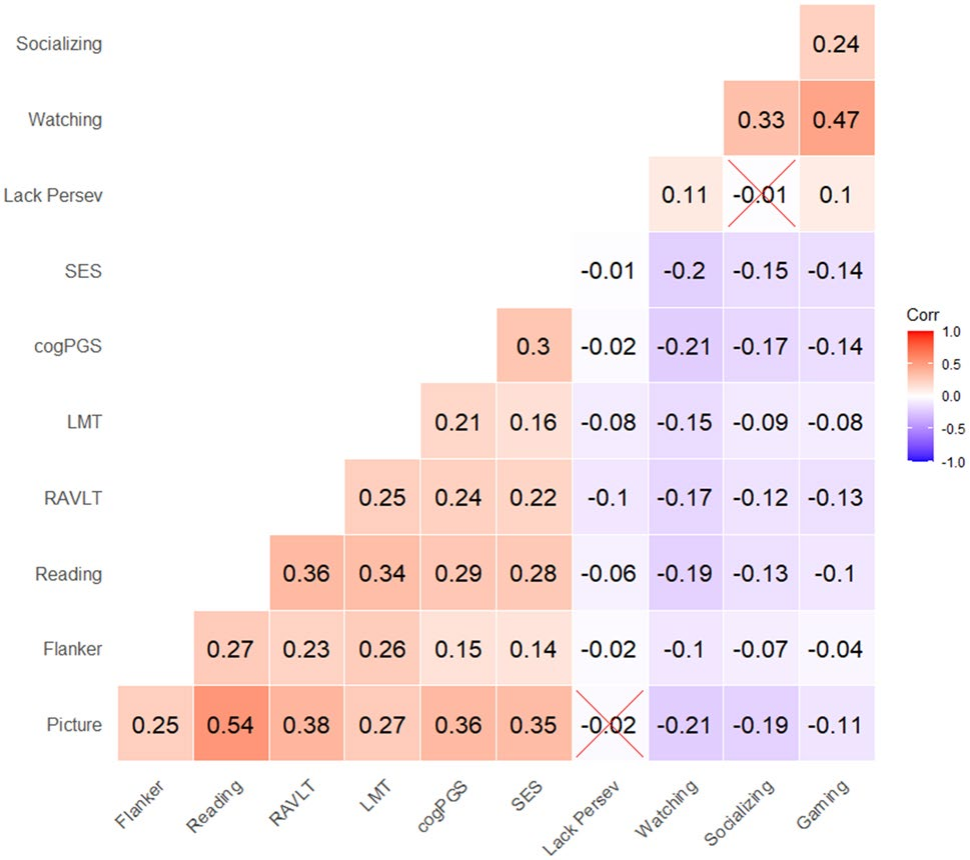
Pairwise correlations between variables at baseline when children were 9–10 years old: all five cognitive tasks, cogPGS (Polygenic scores for cognitive performance), SES (socioeconomic status), lack of perseverance, and all three types of screen time: watching, socializing, and gaming.

Values adjusted for multiple comparison tests with Holm correction. Non-significant correlations are crossed. All other correlations are significant with at least p < 0.05.

Before testing the impact of screen time, we first performed a confirmatory factor analysis of baseline performance in all five cognitive tasks. This model consisted of all five cognitive tasks under the influence of a shared latent factor. The confirmatory factor model fitted the data well, with a root-mean-square error of approximation (RMSEA) = 0.060 and a comparative fit index (CFI) = 0.964, which means that the common variance among all five cognitive tasks is well explained by one latent variable that we call here “intelligence”.

Next, we tested the measurement invariance (also known as measurement equivalence) of our intelligence latent variable over the two years between baseline and follow-up^41^. We found a strong measurement invariance (as shown in “Methods”, a model constraining loadings and intercepts between the two time points was better than models freeing those), with RMSEA = 0.029 and CFI = 0.970. In all subsequent analyses, we used models with strong invariance (fixed loadings and intercepts) between the two time points.

The strong invariance measurement model showed a significant improvement in intelligence after two years from age 9 to 11 (β = 0.24, p < 0.001; or 3.64 IQ points). The amount of change did not depend on baseline intelligence scores at age 9 (r = − 0.05, p = 0.456).

### Latent change score models with cogPGS, SES, and screen time types

We next fitted a model to test the independent impact of screen time after accounting for the effect of cogPGS and SES. Due to the high correlation between the three types of screen time (as seen in Table 1) and their potentially distinct effects on intelligence, the model contained all three types—so the effect of each screen time type on intelligence was also accounted for/controlled by the other two.

**Figure 1:**
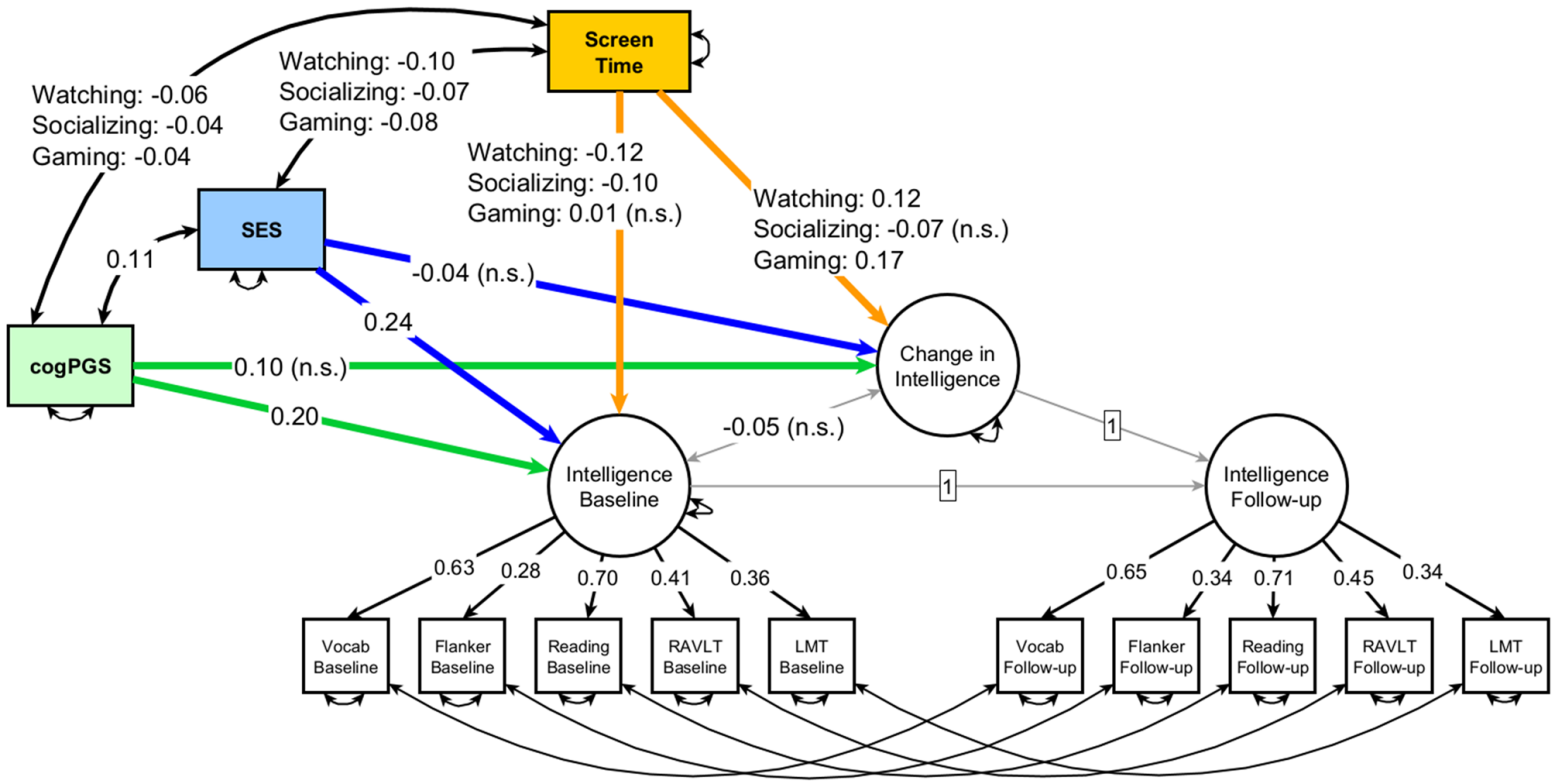
Path diagram of a strict measurement invariant Latent Change Score model with the change in intelligence from ages 9–10 to 11–12. Screen time Watching, Screen time Socializing, Screen time Gaming, cogPGS (Polygenic scores for cognitive performance), and SES (socioeconomic status) are exogenous variables, each already accounting for the effect of the others on baseline intelligence and on the change in intelligence after two years. All variables are standardized. (Which is why the loadings differ between time points—the constraining of loadings and intercepts must be done for the unstandardized estimates.) Non-significant values are marked with “n.s.”. Following convention, rectangles represent observed or exogenous variables and circles represent latent variables (screen time types are shown with only one rectangle for aesthetic reasons—they are actually three distinct rectangles). Single-headed arrows denote regression weights, while double-headed arrows represent variances, covariances, or errors. Standardized betas.

The strong measurement invariant latent change score model (Fig. 1) with the exogenous variables of screen time Watching, screen time Socializing, screen time Gaming, cogPGS, and SES fit the data well: RMSEA = 0.023, CFI = 0.975. Baseline intelligence at age 9–10 had independent, positive associations with cogPGS (β = 0.20, p < 0.001) and SES (β = 0.24, p < 0.001), as well as independent, negative associations with Watching (β = − 0.12, p < 0.001) and Socializing (β = − 0.10, p < 0.001), but no independent association with Gaming (β = 0.01, p = 0.615). All screen time types were negatively associated with both cogPGS and SES.

The follow-up after two years showed that screen time Socializing had no independent effect on the change in intelligence (β = − 0.07, p = 0.220). Interestingly, we found a positive effect on the change in intelligence from screen time Watching (β = 0.12, p = 0.047; or 1.8 IQ points) as well as from screen time Gaming (β = 0.17, p = 0.002; or 2.55 IQ points), with more time watching digital videos or playing video games leading to greater gains in intelligence. As explained above, those results already account for the independent effects of cogPGS (non-significant, β = 0.10, p = 0.069) and SES (non-significant, β = − 0.04, p = 0.871) on the change in intelligence.

In a posthoc analysis suggested by a reviewer, we used only SES’s subcomponent parental education to evaluate how this component in isolation contributed to our main findings. Results with parental education in the main model were mostly the same, but there are a few notable results. The association of parental education to baseline intelligence was a significant 0.32 (while SES in the original model was a significant 0.24). Interestingly, the association between parental education and screen time Watching was a significant − 0.16 (while SES in the original model was a significant − 0.10). Compared to the main model with SES, the model with parental education showed (a now) significant impact of cogPGS on the change in intelligence (β = 0.11, p = 0.047; or 1.65 IQ points) and (a now) nonsignificant impact of screen time Watching on the change in intelligence (β = 0.11, p = 0.068).

In another posthoc analysis, we split the variable Watching into “TV watching” and “online video watching”. We tested the separate effect of these two variables together with Gaming and Socializing in the strong measurement invariant latent change score model. As expected, baseline intelligence at age 9–10 had independent, positive associations with TV watching (β = − 0.09, p < 0.001) and online video watching (β = − 0.07, p < 0.001). Surprisingly, results were different for the change in intelligence. With these two variables split, now there was no significant effect on the change in intelligence from TV watching (β = 0.06, p = 0.246) nor from online video watching (β = 0.09, p = 0.139).

**Figure 2:**
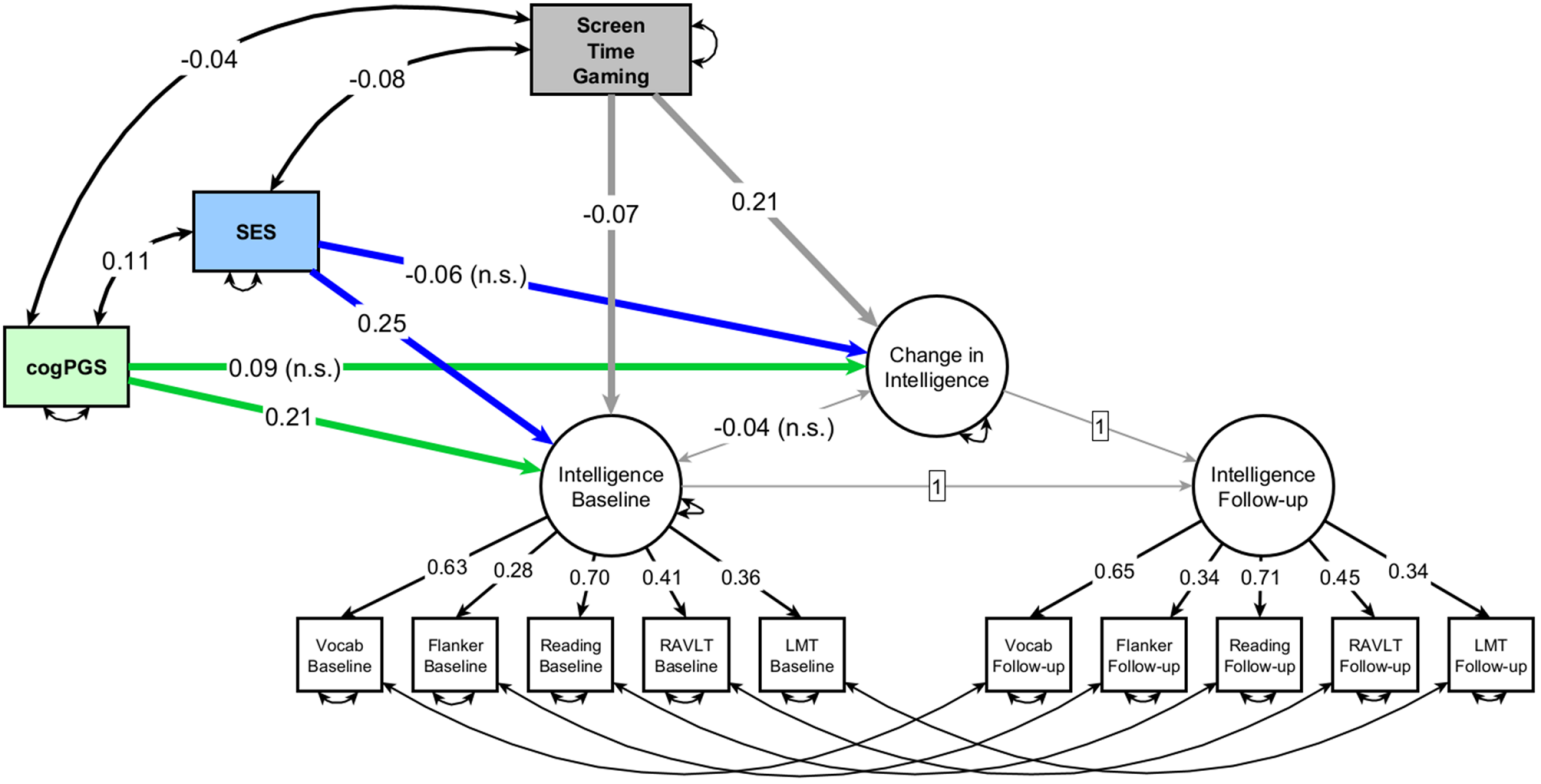
Path diagram of a strict measurement invariant Latent Change Score model with the change in intelligence from ages 9–10 to 11–12. Screen time Gaming, cogPGS (Polygenic scores for cognitive performance), and SES (socioeconomic status) are exogenous variables, each already accounting for the effect of the others on baseline intelligence and on the change in intelligence after two years. All variables are standardized. Non-significant values are marked with “n.s.”. Following convention, rectangles represent observed or exogenous variables and circles represent latent variables. Single-headed arrows denote regression weights, while double-headed arrows represent variances, covariances, or errors. Standardized betas.

Originally, we were also interested in estimating the absolute impact of Gaming, not relative to (or controlled for) the other types of screen time. This had two reasons: (1) a non-relative estimation is typical in the literature, so we can better connect to it; (2) if playing video games has a causal effect, it can be useful to estimate it in an absolute number of hours (so a child Gaming for a small number of hours but who engaged relatively even less in Watching or Gaming will still have a low absolute value for Gaming). The strong measurement invariant latent change score model (Fig. 2) with the exogenous variables of screen time Gaming, cogPGS, and SES fit the data well: RMSEA = 0.025, CFI = 0.972. Of note, baseline intelligence at age 9–10 had an independent, negative association with Gaming (β = − 0.07, p < 0.001). And we found a positive effect on the change in intelligence from screen time Gaming (β = 0.21, p < 0.001), with more time playing video games leading to greater gains in intelligence. (In the Supplementary Information, we also report the separate models fitted with only Watching and with only Socializing).

### Gender differences

**Figure 3:**
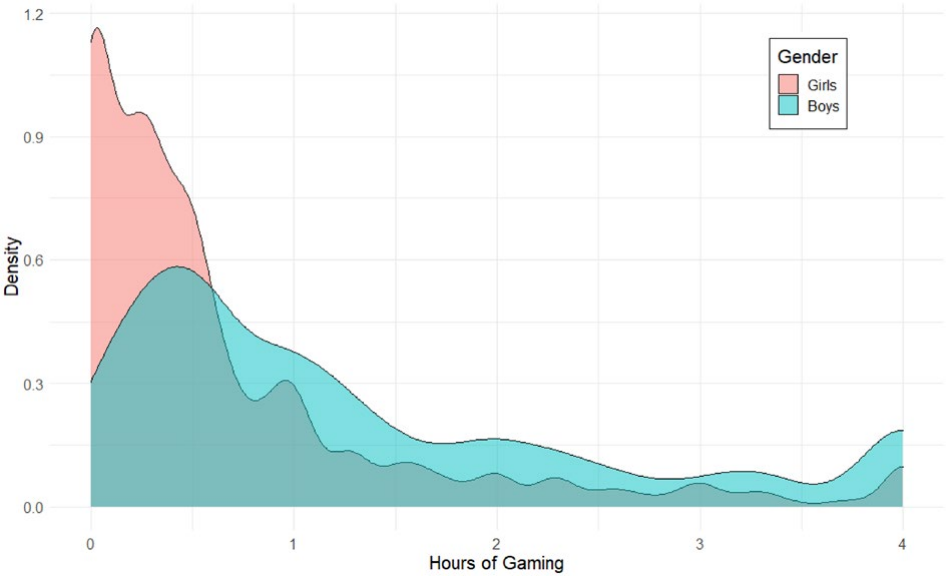
Density plot of time spent Gaming (raw values) between boys and girls at ages 9–10.

Notably, boys on average spent about twice more time Gaming than girls [boys mean = 1.32 (SE = 0.02), girls mean = 0.68 (SE = 0.01)], a difference that was significant and that is illustrated in Fig. 3. Boys also spent slightly (and significantly) more time than girls Watching [boys mean = 2.35 (SE = 0.02), girls mean = 2.18 (SE = 0.03)]. Girls spent significantly more time Socializing [boys mean = 0.47 (SE = 0.02), girls mean = 0.65 (SE = 0.02)].

We determined the role of gender on the associations of cogPGS, SES, and the screen time types with intelligence as well their effect on the change in intelligence. For that, we performed multigroup modeling from above: a strong measurement invariant latent change score model with the exogenous variables of screen time Watching, screen time Socializing, screen time Gaming, cogPGS, and SES. Our multigroup gender analysis showed that a global model with all free parameters between genders had a significantly better fit than a global model with all paths constrained between genders (Chi-square difference = 1093, p < 0.001). Further, we performed our planned gender contrasts of nine specific effects.

The association between screen time and baseline intelligence was not significantly different between boys and girls for Watching (Chi-square difference = 3.76, p = 0.052), or for Socializing (Chi-square difference = 0.84, p = 0.357). That association with baseline intelligence, however, differed for Gaming (Chi-square difference = 6.91, p = 0.008), with no effect in boys (β = − 0.02, p = 0.332) and a positive effect in girls (β = 0.05, p = 0.01), so at age 9–10, girls with higher intelligence tended to play video games slightly more.

For the effect of screen time on the change in intelligence, it was not significantly different between boys and girls for Watching (Chi-square difference = 0.07, p = 0.788) or for Socializing (Chi-square difference = 0.94, p = 0.331), or for Gaming (Chi-square difference = 1.46, p = 0.226).

The effect of baseline intelligence at 9 years old on the change in intelligence after two years was significantly different between boys and girls (Chi-square difference = 8.40, p = 0.004), with a very large positive association in boys (β = 0.64, p < 0.001) and no association in girls (β = − 0.02, p = 0.866). The effect of cogPGS on the change in intelligence was also significantly different between boys and girls (Chi-square difference = 11.59, p < 0.001), with a large positive effect in boys (β = 0.37, p < 0.001) and no effect in girls (β = − 0.01, p = 0.915). Finally, the effect of SES on the change in intelligence was not significantly different between boys and girls (Chi-square difference = 0.30, p = 0.585).

### Mediation by lack of perseverance

To test if the effect of screen time on the change in intelligence after two years was mediated by lack of perseverance, we performed a mediation analysis of the independent, indirect effects of each screen time in the previously used strong measurement invariant latent change score model (exogenous variables of screen time Watching, screen time Socializing, screen time Gaming, cogPGS, and SES). All three mediation models had a good fit (all RMSEA < 0.027, all CFI > 0.968), and showed that lack of perseverance was correlated with cogPGS (all β = − 0.03, all p = 0.003), but not with SES (all p > 0.656). In all three models, lack of perseverance had a negative association with intelligence at age 9 (all β = − 0.08, all p < 0.001), but no effect on the change in intelligence at follow-up 2 years later (all β = 0.08, all p > 0.138).

The mediation model for Watching showed that there was no indirect mediation effect of Watching on the change of intelligence via lack of perseverance (β = 0.01, p = 0.138, CI [− 0.002, 0.012]). The same was true for the other models—no indirect mediation effect of Socializing (β = − 0.001, p = 0.909, CI [− 0.002, 0.002]) or of Gaming (β = 0.008, p = 0.156, CI [− 0.001, 0.002]).

### Sibling analyses

Gene-environment correlations can inflate the estimation of cogPGS on traits^42–44^. Within family effects (*β*_*w*_) are less confounded by the shared environment since the transmission of alleles is random giving each sibling an equal probability of inheriting any given allele. Because of that, a reviewer suggested a posthoc sibling analysis to further test the relationship between screen time and intelligence after accounting for a better estimation of cogPGS (with reduced gene-environment correlations). We estimated the within family direct genetic effects (*β*_*w*_) and the between family genetic effects (*β*_*b*_).

As expected, we found within-family effect of cogPGS on baseline intelligence (*β*_*w*_ = 0.04, p < 0.001) to be noticeably smaller than the between-family effect (*β*_*b*_ = 0.06, p < 0.001). In this model with baseline intelligence, the sibling analysis also revealed that intelligence had associations with Watching (r = − 0.04, p = 0.005) and Socializing (r = − 0.04, p < 0.001), but not with Gaming (r = − 0.001, p = 0.916).

In the sibling analysis with change in intelligence, the within-family effect of cogPGS on the change in intelligence was not significant (*β*_*w*_ = 0.001, p = 0.142) while the between-family effect was significant (*β*_*b*_ = 0.004, p < 0.001). The change in intelligence was not significantly impacted by Watching (β = − 0.0008, p = 0.506), nor by Socializing (β = − 0.001, p = 0.307), nor by Gaming (β = − 0.001, p = 0.182).

## Discussion

Here we estimated the effect of each three types of screen time (*Watching* TV and online videos, *Socializing* via social media, text, and video chat, and *Gaming*) on intelligence after accounting for each other’s screen type, socioeconomic status, and genetic predisposition for intelligence. Our most important finding was that Gaming positively impacted the amount of change in intelligence so that children who played more video games at 9–10 years showed the most gains in intelligence two years later. This was also true for Gaming in absolute values (not correcting for time spent video watching and socializing) and did not differ between boys and girls. Surprisingly, Watching also showed a positive effect on the change in intelligence, and, much less shocking, Socializing had no effect.

### The role of cogPGS and SES

CogPGS and SES showed positive associations with each other and with the baseline measures of intelligence at 9–10 years old, in agreement with past research on children and teenagers^45–47^. CogPGS and SES had no significant impact on the change in intelligence, although our gender analyses revealed that cogPGS had a very large, positive, impact on the change in boys but no effect in girls. Note that accounting for these effect sizes was still important to accurately assess the role of screen time on intelligence. Even non-significant confounders can have a substantial impact^48^—simulations show that a p-value cutoff of 0.20 for confounders identification yielded better accuracy (the p-value of our cogPGS on intelligence change was 0.069), and also suggest that confounders chosen by theoretical reasons (as was the case for both SES and cogPGS here) should be adjusted for regardless of significance^48^.

### Baseline association with screen time

Watching and Socializing were negatively correlated with intelligence at baseline measures when children were 9–10 years old, while Gaming showed no association. These results were mostly in line with a study with the same sample of American children from ABCD^13^. Like here, they found that both Watching activities and Socializing activities were negatively associated with baseline cognition. However, they found a positive association of Gaming with cognition. This difference might be because they used a slightly different set of cognitive tasks (available only at baseline, as their study had no follow-up), and/or, more likely, they did not control for genetic predispositions for cognition.

Note that all measures of video games discussed so far involved time spent playing. A study showed that when expertise in video games is measured, it correlated positively with intelligence^49^.

### The positive effect of gaming and watching on the change in intelligence

Notably, our longitudinal finding of a positive impact of Gaming on the change in intelligence means that children who played more video games were the ones experiencing the most gains in intelligence after two years. This is evidence of a beneficial causal effect of video games on cognition, and, as described before, is supported by multiple different studies. For example, both a correlational and an experimental study found that playing action video games improves visual processing skills and visual attention^50,51^. And a training procedure with action video games found that it increases working memory capacity as compared with training on a control game^52^. In a comprehensive meta-analysis, Powers and collaborators^53^ looked at correlational and quasi-experimental studies comparing video game players with non-players and found that video game players were superior to non-players in measures of executive functioning and other relevant cognitive measures. That study also meta-analyzed randomized experiments and found positive, yet smaller, effects of video game training in all the same measures. Another meta-analysis looked at studies on video gaming in adults and found moderate benefits in overall cognitive ability and moderate to small benefits in specific cognitive domains^54^. Interestingly, younger adults benefited more from video gaming, and this impact was also moderated by the duration and frequency of gaming sessions. And a study by Palaus and collaborators^55^ looked at the neural correlates of video games, and the potential cognitive benefits of playing video games in enhancing attention, verbal and spatial working memory, and visuospatial skills. However, note that there are also negative results in the literature^56,57^.

We also found a positive impact of watching TV and other digital videos on the change in intelligence. This result is much less expected than the results for playing video games. And such a result was also less robust than gaming: our posthoc analysis replacing SES with parental education showed no impact of watching and the change in intelligence (while the impact still existed for gaming). In the model with parental education, there was a much higher negative association of it with screen time. When such a stronger confounder was accounted for in the model, perhaps it was the reason for the lack of impact of watching videos on the change in intelligence. So, the amount of TV shows that children watch is influenced by the education of the parents, and so correcting for it results in a lower estimate. Alternatively, the amount of TV could merely be correlated with parental education and the impact of video watching is spurious (with parental education being the real cause for intelligence changes).

Watching digital content is a fairly passive activity, and the empirical evidence so far suggests a negative impact on cognition from watching TV. For example, a large longitudinal study followed 1000 participants in New Zealand from ages 5 to 13 years and found that more TV time during childhood was associated with symptoms of attention problems in adolescence^58^. That effect remained significant after controlling for gender, socioeconomic status, and cognitive ability in early childhood; and was independent of later television viewing^58^. A review of longitudinal studies in human infants showed that exposure to fast-paced television in the first 3 years of life was linked with attentional deficits in later childhood^59^. The same paper also reviewed experimental studies using mouse models, and these found that excessive sensory stimulation in mice at an early age leads to cognitive deficits^59^. Furthermore, a meta-analysis of 24 cross-sectional studies showed that television viewing was inversely associated with composite academic performance scores, language, and mathematics^3^. Note, however, that some of the studies on video watching aggregate it together with other measures of screen time^60^. The studies that do analyze screen time types separately usually measure data on TV but not on online videos—the latter is arguably much more tailored to the user and can better match their preferences, which could matter to cognitive effects. However, in our current study, we found no evidence for that. Our posthoc analysis separating TV and online videos did not find any difference between these two activities on the change in intelligence.

Perhaps the only solid evidence that supports cognitive benefits from watching digital media is from a recent study in Brazil ^12^. The authors followed up 3800 children from birth and collected cognitive and screen time data (TV, video game, and computer) at ages 11, 15, and 18. The results showed that watching TV (as well as, independently, playing video games, and using/watching on computers) had positive effects on later measures of working memory and intelligence^12^. These effects were already accounting for many potential confounders, such as gender, ethnicity, birth weight, household income, and maternal education^12^. The reader might be left wondering: What could be behind the benefits of passively watching digital content? There is no clear answer to this yet. Arguably, gains in knowledge and cognitive abilities in children could happen because consuming TV and online videos leads to more access to information overall, better quality and better-tailored content from educational videos, and improved technical understanding from navigating/sorting digital content^61^. And there is some empirical support for the idea that high-quality TV/video content (such as the program Sesame Street) has a positive impact on children’s school performance and cognitive abilities^62^. Especially when high-quality TV/video content replaces less enriching activities in the child’s routine^63^.

More profoundly, the positive cognitive impacts of watching videos and playing video games could partially explain the worldwide gradual increase in intelligence scores over the decades: the Flynn effect. This process is known as the “culturo-cognitive” explanation of the Flynn effect via technological progress^64^. Innovations in electronic and information-based technologies over the past decades (i.e., film, television, video games, computers, Internet, etc.) led to iterative and reciprocal processes between the users and designers of technology, whereby generations raised with complex digital media created ever-more-sophisticated technologies that required even greater cognitive skills from the next generation^65^. The end result of this constant informal education creates learners (and therefore intelligence test-takers) with markedly different cognitive skill profiles^64^. These new skills can then be applied by these newer generations in professions that increasingly require those skills. Indeed, this type of iterative and reciprocal development of technologically driven cognitive skills is exactly the kind of process one would expect given the gradual but constant rise in measured intelligence scores over the twentieth century^64^. And it’s probably not a coincidence that the introduction of video games and computer technologies in the 1970s and 1980s coincides with a slight but discernible uptick in the measured rate of increase in IQ scores^66^.

It’s worth mentioning that studies have found a stagnation of the Flynn effect and sometimes even a negative/inverse Flynn effect (gradual reduction in intelligence) in some countries ‒ most notably, developed countries in western Europe. This poses a conundrum to the “culturo-cognitive” explanation because those countries are technologically advanced and have a high prevalence of gaming (such as in Norway^67^). Why the stagnant or even negative/inverse Flynn effect? There are several proposed explanations for that trend, such as maternal age, immigration, dysgenic fertility, and economic decline^68,69^. Though each of these explanations is unlikely to explain the stagnation/reversal of the Flynn effect alone, they could potentially add up in some cases to counteract the gains from watching videos and playing video games.

Although seemingly small, the effects we found of screen time on intelligence would still be practically meaningful. Even seemingly small effects can have critical long-run consequences^70–72^.

### Gender differences and no mediating effect from perseverance

In girls, the ones with higher intelligence scores at ages 9–10 tended to play more video games. In boys, however, there was no such association. And time spent Watching and Socializing showed no gender differences. We found no difference between gender either for the effect of screen time on the change in intelligence after two years. This result is in contrast with a longitudinal study where only men showed an effect of time spent with television and video games (measured when they were 11-years-old) on their later working memory and intelligence at 18 years old^12^. The reasons for this could be due to cultural differences between these two studies—the children in our sample were 9–10 years old in 2017, while theirs were so in 2002.

Notably, we also found that the effect of cogPGS on the change in intelligence had a large positive effect in boys but no effect in girls. Though research on this topic is still rare, our result is in agreement with recent research in which boys tend to have higher polygenic score predictions of educational attainment^73^.

Lack of perseverance did not mediate the effects of screen time on the change in intelligence. This result was somewhat surprising, but perhaps it can be explained because our measure of perseverance is only associated with an unintentional form of interference control rather than with intentionally removing learned material from working memory^74^. Therefore, our measure of lack of perseverance could be less related to intelligence (as shown by the small correlations with cognitive tasks seen in Table 1) and potentially not matter to the role of screen time on the change after two years.

### Sibling analysis

We found the within-family effect of cogPGS to be roughly two-thirds of the between-family effect for intelligence (at baseline), in line with the literature^43,44^. This indicates the presence of passive genotype-environment correlations—whereby parents create family environments consistent with their genotypes, which in turn facilitate the development of their children’s intelligence. In this analysis, the pattern of correlations between baseline intelligence and each type of screen time remained the same as we found in the main models reported above. That is: negative correlations of intelligence with *Watching* and *Socializing*, and no correlation with *Gaming*.

Regarding the change in intelligence, the between-family effect was significant, but the within-family effect was not. Furthermore, none of the screen time types had a significant effect on the change in intelligence. What could account for these discrepancies with the main models reported earlier? Note that the vast majority of our sample did not have siblings, so the sibling analysis is a much smaller subset of the main analyses. For the sibling analysis, we only had data from about 400 families and 1102 individuals (10% of the original sample used in our other analyses). Therefore, a lack of statistical power is most likely the reason for our null findings on the change in intelligence. Another caveat is that in all models reported earlier, cogPGS was estimated with only one member per family (we randomly excluded other siblings to avoid inflating genetic results). But in the sibling analysis, the sample consisted, of course, of all these siblings (all siblings except one of the monozygotic twins, when that was the case).

In light of the sibling analysis’ results, our cogPGS estimate in the full sample and corresponding results on screen time should be interpreted with caution. However, note that research shows SES to be the major source of between-family genetic effects^43,75^. So, the cogPGS in our main model with screen time (without the sibling analysis) is already, to some extent, controlled for these between family gene-environment effects.

### Limitations

Our sample is representative of the United States with respect to sex, race, ethnicity, socioeconomic status, and urbanicity. However, the genome-wide association study^34^ that we used to estimate cogPGS is heavily biased towards those of European descent. This means our results regarding genetics will have much higher accuracy and generalizability in white populations. Another limitation of our study is that we used self-reported measures of screen time. A recent meta-analysis has found that self-reported media use correlates only moderately with logged measurements and that self-reports are a somewhat inaccurate reflection of logged (true) media use^76^.

Furthermore, within the ABCD study, the time spent gaming is described in one variable, including all types of games from very simple smartphone games to complicated multiplayer action games on different consoles. However, from previous studies we know that effects on cognition depend on genres played, skills required, etc.^25,77^. Because children in different countries differ in their preferences for video game genres (https://www.statista.com/statistics/371020/consumer-preferred-video-game-types/), generalization from our results in the USA to other countries is limited.

## Methods

### Study description

The Adolescent Brain and Cognitive Development (ABCD) is a large longitudinal dataset in the US that gathered multiple measures on biology, psychology, and social context in children across 21 research sites. For more information on ABCD protocols and inclusion/exclusion criteria see Volkow et al.^78^. To be included in the present study, participants had to have all the relevant data available: demographics (age, gender, and SES), cognitive tasks, SNP genotypes, and screen time. We used two different time points: the baseline when children were ages 9–10 ended with a final sample of 9855 participants (*M* = 9.90 years, *SD* = 0.62 years, 4686 females), and the follow-up two years later when the children were 11–12 years old had a sample of 5374 participants (*M* = 11.92 years, *SD* = 0.63 years, 2512 females). Note that the follow-up sample is much smaller than the baseline sample because the ABCD collection is still underway and so far, has released only half of the total sample in this follow-up at 11–12 years old.

The research protocol as well as all methods and data collection were reviewed and approved by the central Institutional Review Board (IRB) at the University of California, San Diego. All research was performed in accordance with relevant guidelines and regulations. Written informed consent was obtained from all participants and/or their legal guardians. For more information on the ethical procedures in the ABCD dataset, see Clark et al.^79^.

### Youth screen time survey

The participating children were asked to rate the time they use the following applications on a typical weekday and a typical weekend day: (1) Watch TV shows or movies, (2) Watch videos (e.g., YouTube), (3) Play video games on a computer, console, phone, or another device (e.g., Xbox, PlayStation, iPad), (4) Text on a cell phone, tablet, or computer (e.g., GChat, Whatsapp), (5) Visit social networking (e.g., Facebook, Twitter, Instagram), and (6) Use video chat (e.g., Skype, Facetime). The rating scale ranged from none, < 30 min, 30 min, 1 h, 2 h, 3 h, to > 4 h. We calculated the means of a typical weekday and a typical weekend day.

According to previous findings and reviews^3,80^, contrasting the effects of screen time types, we analyzed major screen-based activities separately. Following the steps of a previous study with this same dataset (measuring baseline associations only^13^, we categorized screen time here into three major groups: (a) Watching (1 + 2), (b) Socializing (4 + 5 + 6), (c) Gaming (3).

### Intelligence

The ABCD dataset has performed a set of cognitive tasks and created indices of crystallized intelligence and fluid intelligence. However, some of the tasks of those indices were not part of the follow-up data collection. Because of that issue, here we created an index of intelligence based on the remaining tasks and added two new relevant cognitive tasks that were available at both time points. The tasks we used here were: Picture Vocabulary Task, Flanker Task, Oral Reading Recognition Task, Rey Auditory Verbal Learning Task, and Little Man Task. For details on each task, see Luciana et al.^81^.

- The Toolbox Picture Vocabulary Task (here called Vocabulary) measured language and vocabulary comprehension. Children hear audio files of words and are shown four pictures in a square, one of which depicts the concept, idea, or object referenced by the auditorily presented words. The child is asked to touch the picture that matches the word. Computerized adaptive testing was implemented to control for item difficulty and avoid floor or ceiling effects.
- The Toolbox Flanker Task (here called Flanker) assesses participants’ inhibition of surrounding stimuli distracting from the target (arrows). Four flanking arrow stimuli (2 on the outer left and 2 on the outer right) all face the same way, either left or right, and the middle arrow is then either facing the same way (congruent trial) or a different way (incongruent trial). Children have to push a button to indicate whether the middle stimulus is facing left or right.
- The Toolbox Oral Reading Recognition Task (here called Reading) measures language decoding and reading. Children were asked to read aloud single letters or words presented in the center of the screen. Item difficulty was modulated using computerized adaptive testing.
- The Rey Auditory Verbal Learning Task (here called RAVLT) measures auditory learning and memory recall. Participants listened to a list of 15 unrelated words and were asked to immediately recall these after each of five learning trials. The total number of items correctly recalled across the five learning trials was summed to produce a measure of auditory verbal learning.
- The Little Man Task (here called LMT) was used to test visual-spatial processing, including mental rotation. During the task, a male figure holding a briefcase in his right or left hand is presented. The figure is shown in one of four positions; facing the respondent, with his back to the respondent, right side up, or upside down. Participants should indicate in which hand the man holds the briefcase, via button press. The task includes practice trials and 32 assessment trials. Performance is represented by percent correct in all 32 assessment trials.

### Socioeconomic status and parental education

Socioeconomic status (SES) was defined as the first principal component from a probabilistic PCA of total household income, parental education, and neighborhood quality. We used a probabilistic PCA because of decent amounts of non-overlapping missing data in parental income and neighborhood quality. Children missing more than one of the three measures were excluded (n = 45). Parental education was recoded to reflect middle school or less (1), some high school (2), high school graduate (3), some college/associates degree (4), bachelor’s degree (5), a master’s degree (6), or professional degree (7). Our measure of neighborhood quality was the area deprivation index calculated from the American Community Survey using the address of primary residency^82^. The SES composite and each subcomponent were standardized with a mean of zero and a standard deviation of one.

As suggested by a reviewer, we also performed a post hoc analysis of our main model using only the subcomponent of parental education. Recent research has highlighted issues and limitations arising from using SES as a composite measure^83^. It might be that parents’ education is the subcomponent that matters in shaping children’s intelligence^84,85^, so its impact could be diluted/contaminated when combining it with income and neighborhood quality.

### Lack of perseverance

Lack of perseverance was measured as part of the ABCD’s abbreviated UPPS-P-Youth Version. The measure comprised 4 items rated on a 1 (agree strongly) to 4 (disagree strongly) scale. The four items for lack of perseverance were: “I finish what I start”, “I tend to get things done on time”, “I am a person who always gets the job done”, and “I almost always finish projects that I start”. For more details, see Watts et al.^86^.

### Genetics

Genotyping was done by the ABCD study, and the data was provided to us. Saliva samples were collected at the baseline visit. The genotyping was performed using the Smokescreen array^87^, consisting of 646,247 genetic variants.

Quality control (QC) and imputation were performed by the National Bioinformatics Infrastructure Sweden (NBIS), as a service contracted by us. Before imputation, SNPs were excluded if they had high levels of missing data (SNP call rate < 98%), departed from Hardy–Weinberg equilibrium as calculated in the lfa R package (sHWE) (P < 1 × 10 − 6), or had minor allele frequencies (MAF) < 1%. Moreover, individuals with an absolute autosomal heterozygosity > 0.2 or more than 2% missing genotypes were excluded. These filtering steps resulted in a cleaned dataset of 10,069 individuals and 430,622 variants. Subsequently, haplotypes were pre-phased with SHAPEIT2. Genetic markers were imputed using the IMPUTE4 software and the 1000 Genomes References Panel (phase 3, build 37). After imputation, genotypes with an INFO score < 0.3 or a MAF < 0.001% were excluded. The final number of SNPs after imputation was 40,637,119 in a total of 10,069 individuals. To check for outliers and to control for population structure the PCA module as implemented in RICOPILI was used. We used the first 20 principal components from that genetic PCA, as described below.

After that, our group then created polygenic scores for cognitive performance (here called “cogPGS”) for each participant using PRSice-2^88^. This was calculated by the sum of effect sizes of thousands of SNPs (weighted by how many of the effect alleles were present in each individual) that were discovered by a large genome-wide association study on educational attainment, mathematical ability, and general cognitive ability^34^. That study has available all effects sizes and p values of their SNPs on the website of the Social Science Genetics Association Consortium (https://www.thessgac.org/data). We used the data available by the consortium from a multi-trait analysis of GWAS^89^, which, in our case, represents a joint polygenic score focused on a GWAS of cognitive performance and complemented by information from a GWAS on educational attainment, a GWAS on the highest-level math class completed, and a GWAS on self-reported math ability. This joint analysis is ideal because pairwise genetic correlations of these traits were high^34^. Furthermore, these GWAS had hundreds of thousands of individuals, and such a large sample size allows new studies to detect effects in samples of a few hundred individuals with 80% statistical power^34^.

For the creation of cogPGS in our samples, we performed clumping and pruning to remove nearby SNPs that are correlated with one another. The clumping sliding window was 250 kb, with the LD clumping set to r^2^ > 0.25. We included the weightings of all SNPs, regardless of their p-value from the GWAS (p = 1.00 threshold). At the end of this process, we had 5255 SNPs included. We standardized the cogPGS to have a mean of zero and a standard deviation of one.

### Statistical analysis

For all analyses, we used R version 4.0.3^90^ and, notably, the packages: lavaan, semTools, lme4, and pcaMethods.

The ABCD dataset contains siblings and twins and this information could erroneously alter variance and inflate the precision of our study. Therefore, we kept only one participant at random from each family. Then, each original variable used in the models was put in linear regressions with the 20 first principal components of the SNP genotype data to correct for population stratification (associations of environmental effects with genetic structure due to geographic separation, population mixtures, and migrations that occurred throughout our evolutionary history)^91,92^, as well as with the age of the participants at baseline and at the follow-up to correct for age differences, and with the site of the participants to correct for collection site differences. From these regressions, we obtained standardized residuals for all variables at baseline and follow-up and used them in all subsequent analyses. As by the Frisch–Waugh–Lovell theorem, such prior covariate residualization from all relevant exogenous and endogenous variables is equivalent to running a regression model with all covariates present (which we couldn’t do here since structural equation modeling cannot handle our large number of covariates).

We chose to use a latent change score model as it allowed us to examine the effect on intelligence from each type of screen time, cogPGS, and SES simultaneously and with reduced measurement error^93,94^. The model also allowed us to estimate the effect of screen time at both baseline values of intelligence at age 9–10 as well as the change in intelligence over two years even after accounting for genetic effects. A latent change score model can be conceptualized as a reparameterization of a paired t-test and has recently been highlighted for its usefulness in teasing apart the complex processes involved in longitudinal developmental research^95,96^. We estimated a latent change score model on the change in intelligence from ages 9–10 to 11–12 and containing the predictors (exogenous variables) of cogPGS (Polygenic scores for cognitive performance), SES (socioeconomic status) and all screen time variables: Watching, Socializing, and Gaming. We also estimated models for each screen time variable separately (as reported in the Supplementary Information). For model estimation, we used full-information maximum likelihood and a robust maximum-likelihood estimator with a Yuan–Bentler scaled test statistic from the R package lavaan version 0.6–9^97^. We assessed model fit using the comparative fit index, (CFI fit > 0.95), and the root-mean-square error of approximation (RMSEA fit < 0.08)^98^.

Before fitting the latent change score model, we assessed fit on respective measurement models, as well as tested for measurement invariance. We used the method of measurement invariance described by Putnick and Bornstein^41^. This allowed us to assess the psychometric equivalence of a construct across time. We found that our measurement across baseline and follow-up had strong measurement invariance (fixed loadings and fixed intercepts from the cognitive tasks between time points), RMSEA = 0.029 and CFI = 0.970. The metric model (fixed loadings) was better than the configural model (same variables between time points) with a CFI change of 0.005, RMSEA change of 0.006, and SRMR change of − 0.004; following the cutoff criteria suggested by Putnick and Bornstein^41^. That means that each cognitive task has the same contribution to our latent construct of intelligence across the two time points—in other words, the construct of “intelligence” has the same meaning at baseline and two years later at follow-up. Similarly, the scalar model (fixed intercept) was better than the metric model (fixed loadings) with a CFI change of 0.01, RMSEA change of 0.001, and SRMR change of − 0.004. That means that the mean differences in our latent construct of intelligence across timepoints capture the mean differences in the shared variance of the cognitive tasks—so, participants who have the same value on the latent construct of intelligence should have equal values on the individual cognitive tasks. Given these results, in all subsequent analyses, we used models with strong invariance (fixed loadings and intercepts) between the two time points.

To test the role of gender differences in our study, we performed a multigroup analysis with the main model described above. We started with the global estimation of two models: one in which all parameters are allowed to differ between groups, and one in which all parameters are fixed to those obtained from analysis of the pooled data across groups. We call the first model the “free” model since all parameters are free to vary and the second the “constrained” model since each path, regardless of its group, is constrained to a single value determined by the entire dataset. If the two models are not significantly different (based on a Chi-squared difference test), and the latter fits the data well, then there is no variation in the path coefficients by gender and a multigroup approach is not necessary. But if it is significant, we can undergo the process of introducing and releasing constraints to try and identify which path varies between groups. We had 9 planned contrast tests: the paths from screen time to baseline intelligence and to the change in intelligence (one for each type of screen time, so 6 contrasts for gender) plus the path from baseline intelligence to the change in intelligence, the path from cogPGS on the change in intelligence, and the path from SES on the change in intelligence.

Finally, we also tested a potential meditation of screen time on the change in intelligence via lack of perseverance. For that, we used the method of SEM mediation and performed 1000 bootstrap runs for bias-corrected confidence intervals^99^. We followed modern recommendations on mediation analyses^100^. The SEM mediation allowed us to test the indirect effect itself by product-of-paths (or difference in coefficients) approach, which cannot usually be done in traditional, regression frameworks.

In a posthoc analysis suggested by a reviewer, we conducted a sibling analysis using the procedure described by Selzam et al.^43^. Such analysis allowed us to compare members (twins and siblings) of the same family and further test the relationship between screen time and intelligence. We estimated the within family direct genetic effects (*β*_*w*_) and the between family genetic effects (*β*_*B*_). For families to be included in the analysis, they needed to have at least two non-identical siblings. We performed mixed effect models for baseline intelligence and change in intelligence. We included the covariates of age, site, 20 PCAs of ancestry, and SES.

## Supplementary Information

Supplementary Information.
