# Supplementary material for: The impact of digital media on children’s intelligence while controlling for genetic differences in cognition and socioeconomic background
Source: Sci Rep. 2022 May 11;12:7720. doi: 10.1038/s41598-022-11341-2 (PMC9095723; doi:10.1038/s41598-022-11341-2)
Supplement: Supplementary file 1 — Supplementary Information. [file 41598_2022_11341_MOESM1_ESM.docx]

**Supplementary Information**

Results of the models per type of screen time variable

- Screen time: Watching

A strong measurement invariant latent change score model with the exogenous variables of screen time Watching, cogPGS, and SES (Figure SI 1) fitted the data well: RMSEA = 0.024, CFI = 0.974. Screen time Watching was negatively associated with both cogPGS (r = -0.06, p < 0.001) and SES (r = -0.10, p < 0.001). Screen time Watching had a negative association with intelligence at age 9-10 (β = -0.15, p < 0.001), even with the model already accounting for the effects of cogPGS (β = 0.20, p < 0.001) and SES (β = 0.24, p < 0.001) in baseline intelligence. The follow-up after two years showed screen time Watching independently affecting the change in intelligence in the positive direction (β = 0.18, p = 0.001), with more time spent watching videos led to larger gains in intelligence. That result already accounts for the independent effects of cogPGS (non-significant, β = 0.10, p = 0.067) and SES (non-significant, β = -0.05, p = 0.342) on the change in intelligence.


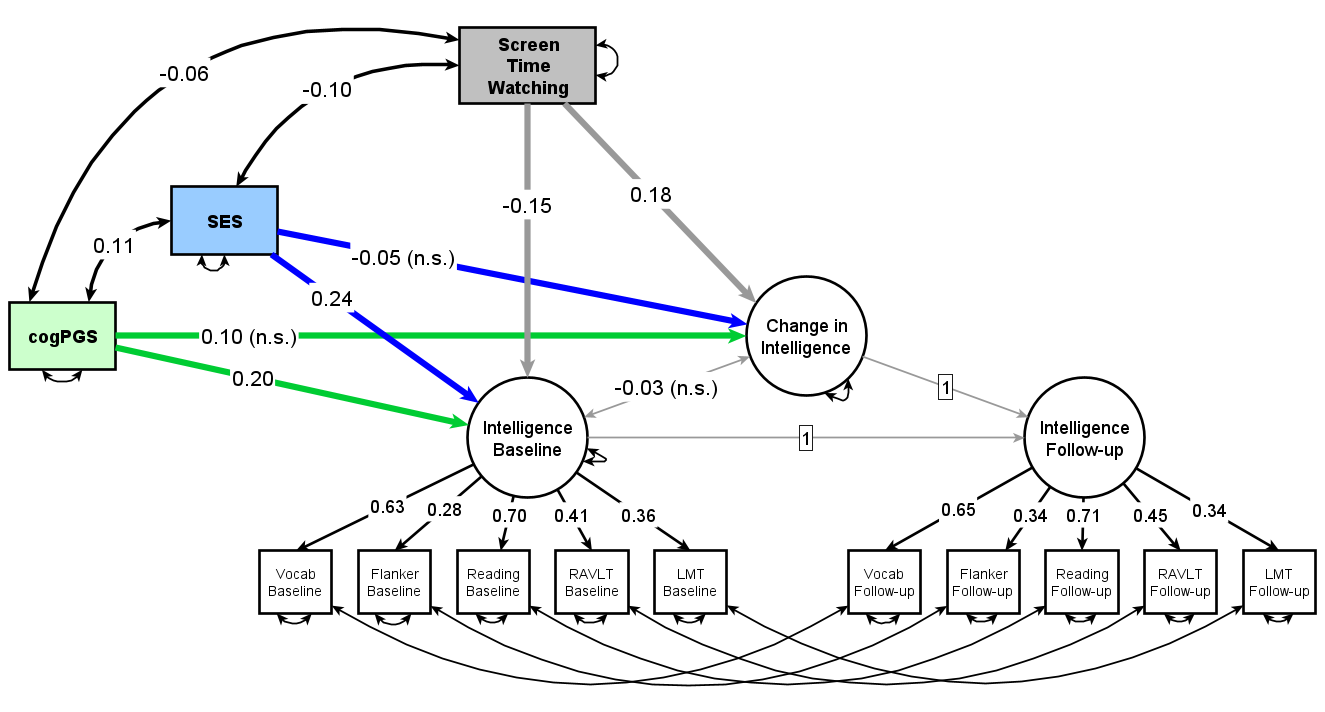


Figure SI 1. Path diagram of a strict measurement invariant Latent Change Score model with the change in intelligence from ages 9-10 to 11-12. Screen time Watching, cogPGS, and SES are exogenous variables, each already accounting for the effect of the others on baseline intelligence and on the change in intelligence after two years. All variables are standardized. Non-significant values are marked with “n.s.”. Following convention, rectangles represent observed or exogenous variables and circles represent latent variables. Single-headed arrows denote regression weights, while double-headed arrows represent variances, covariances, or errors.

- Screen time: Socializing

A strong measurement invariant latent change score model with screen time Socializing, cogPGS, and SES as exogenous variables (Figure SI 2) fitted the data well: RMSEA = 0.024, CFI = 0.973. Screen time Socializing was negatively associated with both cogPGS (r = -0.04, p < 0.001) and SES (r = -0.07, p < 0.001). Screen time Socializing had a negative association with intelligence at age 9-10 (β = -0.13, p < 0.001), even with the model already accounting for the effects of cogPGS (β = 0.20, p < 0.001) and SES (β = 0.25, p < 0.001) in baseline intelligence. In the followup after two years, screen time Socializing was not independently related to change in intelligence (β = -0.01, p = 0.933). That result already accounts for the independent effects of cogPGS (non-significant, β = 0.09, p = 0.091) and SES (non-significant, β = -0.06, p = 0.258) on the change in intelligence.


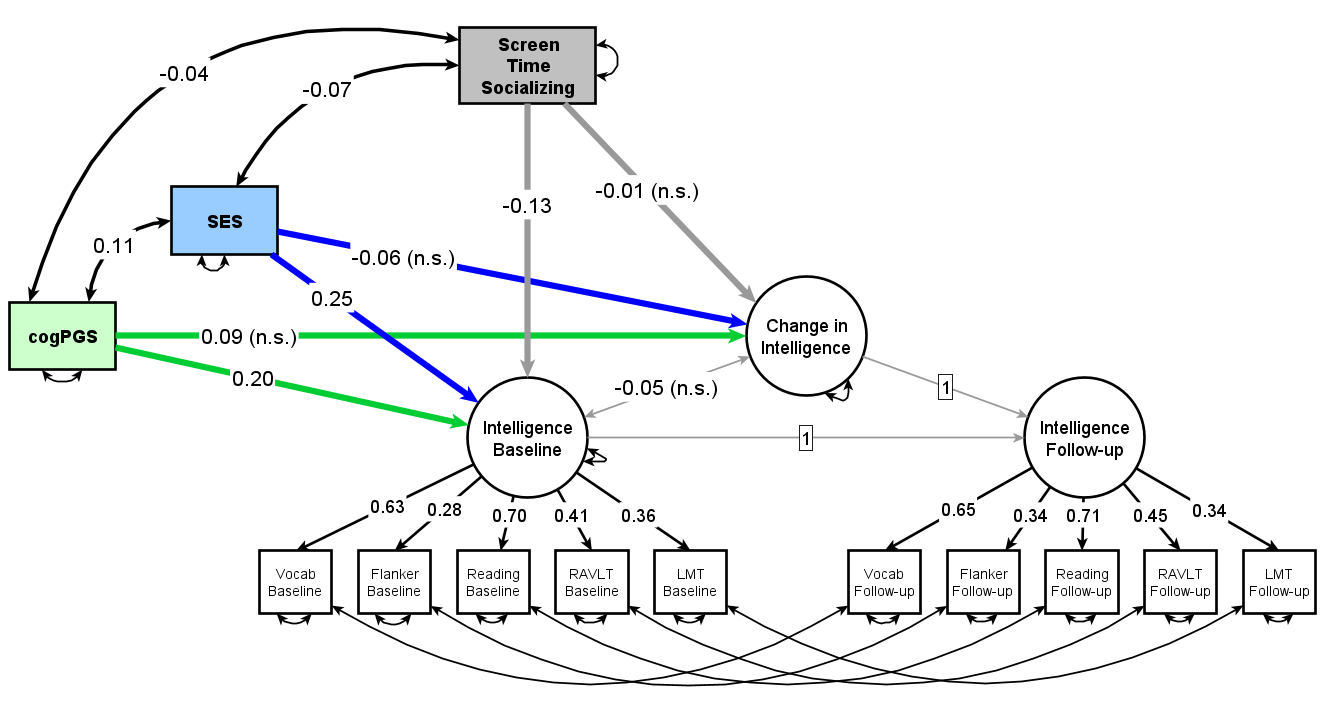


Figure SI 2. Path diagram of a strict measurement invariant Latent Change Score model with the change in intelligence from ages 9-10 to 11-12. Screen time Socializing, cogPGS, and SES are exogenous variables, each already accounting for the effect of the others on baseline intelligence and on the change in intelligence after two years. All variables are standardized. Non-significant values are marked with “n.s.”. Following convention, rectangles represent observed or exogenous variables and circles represent latent variables. Single-headed arrows denote regression weights, while double-headed arrows represent variances, covariances, or errors.

- Screen time: Gaming

A strong measurement invariant latent change score model with the exogenous variables of screen time Gaming, cogPGS, and SES (Figure SI 3) fitted the data well: RMSEA = 0.025, CFI = 0.972. Screen time Gaming was negatively associated with both cogPGS (r = -0.04, p < 0.001) and SES (r = -0.08, p < 0.001). Screen time Gaming had a negative association with intelligence at age 9-10 (β = -0.07, p < 0.001), even with the model already accounting for the effects of cogPGS (β = 0.21, p < 0.001) and SES (β = 0.25, p < 0.001) in baseline intelligence. Interestingly, the follow-up after two years showed screen time Gaming independently affecting the change in intelligence in the positive direction (β = 0.21, p < 0.001), with more time spent playing video games leading to larger gains in intelligence. That result already accounts for the independent effects of cogPGS (non-significant, β = 0.09, p = 0.076) and SES (non-significant, β = -0.06, p = 0.338) on the change in intelligence.


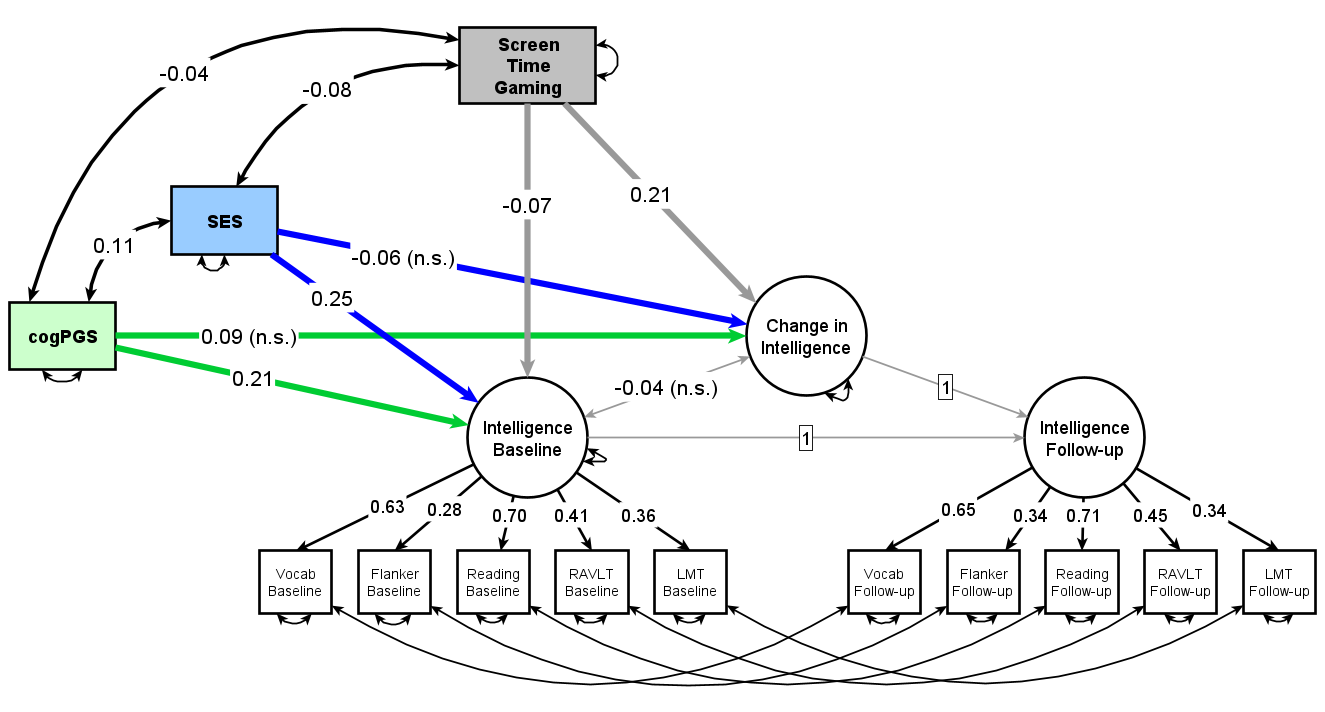


Figure SI 3. Path diagram of a strict measurement invariant Latent Change Score model with the change in intelligence from ages 9-10 to 11-12. Screen time Gaming, cogPGS, and SES are exogenous variables, each already accounting for the effect of the others on baseline intelligence and on the change in intelligence after two years. All variables are standardized. Non-significant values are marked with “n.s.”. Following convention, rectangles represent observed or exogenous variables and circles represent latent variables. Single-headed arrows denote regression weights, while double-headed arrows represent variances, covariances, or errors.
